# Infected Non-union of Tibia Treated with Ilizarov External Fixator: Our Experience

**DOI:** 10.5704/MOJ.1903.006

**Published:** 2019-03

**Authors:** S Fahad, AA Habib, MB Awais, M Umer, HU Rashid

**Affiliations:** Section of Orthopaedics, Aga Khan University Hospital, Karachi, Pakistan

**Keywords:** non-union of tibia, infection, Ilizarov technique, bony union

## Abstract

**Introduction:** Tibia is the most common long bone fractured due its vulnerable subcutaneous location and most often associated with acquired complications of delayed union or non-union due to infection. Amongst the various treatment options to treat them, the Ilizarov external fixator application is considered superior due to its multiple advantages. The objective of this study was to analyse the role of Ilizarov fixation in infected tibial non-union, as well as to assess bony union and associated functional outcomes.

**Materials and Methods:** A retrospective review was conducted for the duration between 1st January 2005 to 31st December 2016. Total of fifty-one patients with tibial non-union associated with infection who treated with the Ilizarov fixator were included in the study. Patient records were reviewed for union of bone, bone and functional outcomes and complications.

**Results:** The most common organism for infection was identified to be *Staphylococcus Aureus*. At the time of final follow-up all patients had achieved union except two, one of whom had to undergo amputation due to non-union and sepsis. Majority of the patients had an excellent score as per ASAMI grading system for bone and function results. The most common complication noted was pin track infections.

**Conclusion:** In our experience, Ilizarov external fixator is better suited for infected non-union of tibia because it can provide a stable mechanical environment, bone transport, correct deformities, and enable weight bearing and hence we recommend its use for the same.

## Introduction

The incidence of complex and compound fractures of long bones is on an increasing trend due to increasing number of high energy trauma events in recent times^1^. Tibia is the most common long bone fractured due its vulnerable subcutaneous location. Delayed union and non-union due to infection are some of the commonly acquired complications^2^. Non-union is more common in tibia fractures compared to other bones of the body.

Additionally, non-union of fracture is often complicated by other coexisting problems, such as persistent infection, loss of soft tissues and bone, limb length discrepancy and limb deformity^3^. Infected tibial non-union has always posed a challenge for orthopaedic surgeons^4^. There are different options available for the management of chronic diaphyseal infections associated with non-union which include extensive debridement with local soft-tissue rotational flaps^5^, packing the defect with antibiotic cement beads, Papineau-type open cancellous bone grafting^6^, tibiofibular synostosis, free microvascular soft-tissue and bone transplants and the Ilizarov method. The Ilizarov method has certain advantages as it can overcome most of the difficulties; it can compensate for bony defects, allow for bony union through bone histogenesis as well as eliminate infection^7^.

The primary aim of this study was to analyse the role of Ilizarov fixation in infected tibial non-union, as well as to assess infection rates, bony union, functional outcomes and associated complications.

## Materials and Methods

From 1st January 2005 to 31st December 2016, the hospital records of fifty-one patients treated for infected non-union of tibia with Ilizarov technique were reviewed retrospectively. Inclusion criteria for the study were tibial non-union of minimum duration of six months and infection at the site of non-union with an additional criterion of either a bone defect of more than 2.5cm or an attempt to attain bony union that failed to heal following an intervention, for an example by doing exchange nailing or bone grafting. Tibial non-union not associated with infection and infected fractures of less than six months duration were excluded from study. All procedures were performed by fellowship trained surgeons with at least five years experience in Ilizarov application.

Demographic details, the cause of initial injury, number of previous operations, the type of previous internal or external fixation and the organisms isolated were noted. Non-union was classified according to the infection being either active or quiescent, and the amount and extent of bone loss. Peri-operative complications were assessed and the details for any additional procedures were compiled using a proforma.

The initial treatment of the fracture had been open reduction and internal fixation (ORIF) in 17 patients, external fixation in 14 patients, intramedullary nailing in 11 patients, and cast application in seven patients. Two patients with Ilizarov application as the primary choice of treatment for the fracture had presented with development of non-union. The mean number of previous surgeries was two (range: 0-14).

The patients were positioned supine on a radiolucent table. Ilizarov fixator was assembled with respect to patient’s limb length, site of infected non-union and functional status of the ankle and knee joints. Following this, the incision, the scope of resection and the pre-selected osteotomy site were marked to complete the preparation for surgery.

The assembled Ilizarov fixator was applied at the tibial shaft in a manner that the Ilizarov rings were positioned on the proximal and distal fragments parallel to the respective joints and the pins were inserted into the same plane keeping them perpendicular to the mechanical axis of the tibia under image intensifier control. This step was very critical for the procedure and failure to perform it before resection of the infected bone could result in the reduction becoming very difficult due to loss of reference object. The operative incision was then made in accordance with the incision marked beforehand.

Radical debridement was carried out for the necrotic bone and infected soft tissue. Bone ends having bleeding margins were considered as vital bones. Fibular osteotomy with resection of fibula segment was done via a sub-periosteal transverse osteotomy where limbs were complicated with deformity or shortening.

Post-operatively, a course of appropriate intravenous antibiotics was given for two weeks to all patients according to culture and sensitivity. Patients in whom culture was negative were treated with four weeks of broad spectrum of antibiotics covering gram positive and gram negative organisms. Full weight bearing using crutches and isometric exercises as well as those for range of motion were encouraged from the first post-operative day. The latency period prior to bone transport was 5-7 days, while the distraction rate was 0.25mm per 6 hours. After completion of bone transport, the tibia docked ends were compressed by 0.25mm per day to provide full contact until the patient felt pain at the docking site.

**Fig. 1:**
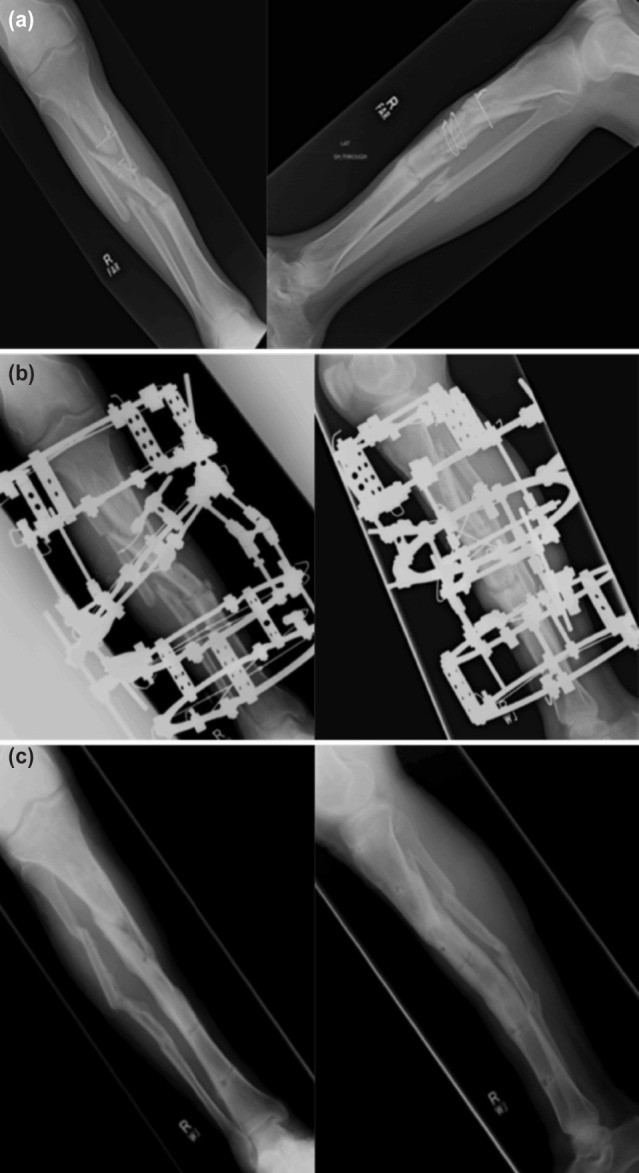
Radiographs of Patient 1. (a) Pre-operative antero-posterior and lateral views showing tibial non-union. (b) Post-operative antero-posterior and lateral views after Ilizarov application. (c) Antero-posterior and lateral views at the time of final follow-up showing bony union.

**Fig. 2:**
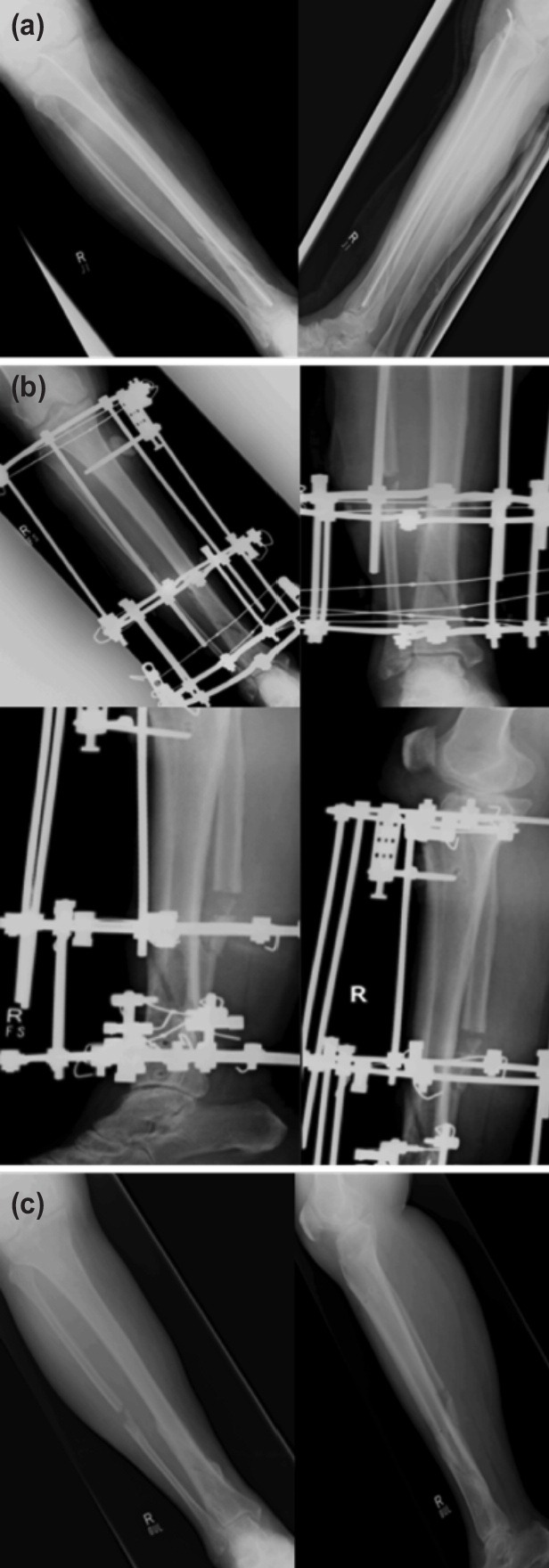
Radiographs of Patient 2. (a) Pre-operative antero-posterior and lateral views showing tibial non-union. (b) Post-operative antero-posterior (top row) and lateral (bottom row) views after Ilizarov application. (c) Antero-posterior and lateral views at final follow-up showing bony union.

Time for external fixation and bone transport, external fixation index and any observed complications were recorded. Radiographs were repeated and analysed every two weeks during the distraction period and every month during the consolidation period. The pre-and post-operative radiographs and at final follow-up of two patients treated are shown in Figures 1 and 2. Removal of Ilizarov fixator was planned when there was evidence of solid docking site union and the regenerated area had at least three complete cortices. Objective evaluation of bone and functional results were done using Association for the Study and Application of the Method of Ilizarov (ASAMI) classification^8^.

## Results

A total of fifty-one patients were included in the study. Mean age of the study population was 45.65±16.69 years. Fourty-one patients (80%) were males and 10 patients (20%) females. Mean follow-up period was 36.84 months (range: 3-45 months) while the mean Ilizarov fixator time was ten months. Road traffic accident was the most common mechanism of injury in patients presenting with tibial non-union followed by fall, gunshot and blast injury (Table I).

**Table I:** Mechanism of initial injury

Mechanism of initial trauma	Frequency	Percentage
Road traffic accident	33	64.7%
Fall form height	10	12.62%
Gun shot	6	8.73%
Blast injury	2	3.9%

Cultures were positive in 27 patients (54%) and *staphylococcus aureus* was the most common organism isolated (Table II). Single organism was grown on cultures from 21 patients whereas two patients had cultures with growth of two organisms and four patients with three organisms.

**Table II:** Organisms isolated from culture

Organisms (n=27)	Number
Staphylococcus aureus	17 (33.3%)
Escherichia coli	3 (5.9%)
Pseudomonas aeruginosa	6 (11.8%)
Proteus mirabilis	1 (2.0%)

Fourty-nine patients were able to bear weight till the last follow-up while only one patient had difficulty in weight bearing. One patient unfortunately had to undergo amputation because of non-union and sepsis. Out of all the patients, 13 patients still complained of pain on weight bearing whereas 38 patients were free of pain at the time of their last follow-up. The mean bone defect was 3.5 (range: 2-5) cm. The mean external index was 60 days/cm (range: 45-120days/cm). Eight patients had soft tissue defect that require soft tissue coverage. In seven patients local flap were used while free flap was used in one patient. The mean surgical time was 180 minutes (range: 120-300minutes). Pre-operative limb length discrepancy was present in 30 patients which was corrected in 23 patients, while seven patients had residual limb length discrepancy, of whom five had less than 2cm leg length discrepancy. Eradication of infection, both clinically and radiologically, was achieved in 50 patients. According to ASAMI grading system for bone, 22 patients had excellent, 19 good, seven fair and three had poor results.

**Fig. 3:**
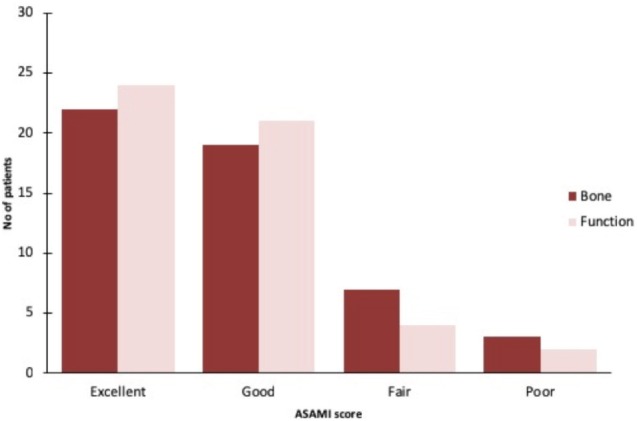
Comparison of outcomes as per ASAMI scoring system.

At the last follow-up, 49 patients were able to bear weight fully on the affected leg, without the use of any crutch or walking aid. Thirteen patients had pain on walking when performing activities of daily living. Eight patients had an obvious limp at last follow-up but only one of these patients had an impairment in performing activities of daily living. The functional result was excellent in 24 patients, good in 21, fair in five and poor in one (Fig. 3, Table IV). No peri-operative complication was observed while nine patients had post-operative pin-track infections, treated with antibiotics. Wire loosening and non-union occurred in two patients each. Reinfection, leg abscess and septic arthritis occurred in one patient each. None of the patients expired during the treatment process (Table III).

**Table III:** Complications observed after ilizarov

Complications	Frequency
Pin track infection	9 (17.6%)
Non-union	2 (3.9%)
Wire loosening/broken	2 (3.9%)
Re-infection	1 (2.0%)
Leg abscess	1 (2.0%)
Schanz Screw broken	1 (2.0%)
Septic arthritis	1 (2.0%)
Limb length discrepency	7 (14.2%)

**Table IV:** Bone and functional outcomes using Association for the Study and Application of the Methods of Ilizarov (ASAMI) system

Bone results	Criteria	Number of patients
Excellent	Union, no infection, deformity < 7°, limb-length discrepancy < 2.5 cm	22
Good	Union + any two of the following: absence of infection, < 7° deformity and limb-length inequality of < 2.5 cm	19
Fair	Union + only one of the following: absence of infection, deformity < 7° and limb-length inequality < 2.5 cm	7
Poor	Nonunion/re-fracture/union + infection + deformity > 7° + limb-length inequality > 2.5 cm	3
Functional results	Criteria	Number of patients
Excellent	Active, no limp, minimum stiffness (loss of < 15° knee extension/< 15° dorsiflexion of ankle), no reflex sympathetic dystrophy (RSD), insignificant pain	24
Good	Active, with one or two of the following: limp, stiffness, RSD, significant pain	21
Fair	Active, with three or all of the following: limp, stiffness, RSD, significant pain	4
Poor	Inactive (unemployment or inability to return to daily activities because of injury)	2

## Discussion

Managing non-union with large bony defects can be a challenging problem for orthopaedic surgeons. There are multiple ways of treating them, for instance, with ring fixators, modified arbeitsgemeinschaft für osteosynthesefragen (AO) fixators or specialised intramedullary nails. However, for complex non-unions (defect >4cm) it is seen that Ilizarov fixator provides a more superior method of treatment^9^.

This is the largest retrospective study from Pakistan thusfar regarding the treatment of infected non-union of tibia by the Ilizarov method. We used the ASAMI score to analyse the effectiveness of Ilizarov method in treating such cases. The excellent and good rates of bone result using the ASAMI score came out to a total of 80% (41/51) while that of the functional outcome was 88% (45/51). These values are comparable to the results of the study done by Yin *et al*^10^. In our study, the functional result was better than the bone result differing from most studies of this kind such as Magadum *et al* and Farmanullah *et al*, both of which had a better bone score than the functional score (76%>60% and 58.9%>56.9% respectively)^11,12^. A previous study also demonstrated a similar result as ours with a better functional score than the bone score (64%>60.8%)^13^. This difference could be attributed to the functional score being dependent on a variety of other factors including the patient’s pain threshold and the conditions of the muscles, bones and joints^9^.

More than 96% of the patients achieved bone union while recurrence of infection was observed only in one patient. Similar results were shown in a study conducted by Xu *et al* in which 100% of the patients achieved union while none of the patients developed deep infection as a complication of using Ilizarov technique for infected non-unions of the tibia^14^.

The patients who underwent multiple procedures before Ilizarov application and had a higher time interval between initial trauma and Ilizarov application had less favorable outcomes as opposed to those who underwent a single surgery before the Ilizarov technique and had less time duration between initial injury and Ilizarov application. Previous research also showed that increased interval between injury and surgical intervention resulted in higher rates of infection^15^. On the other hand, there have been multiple studies that contradict this rule such as an extensive literature review done by Crowley *et al* which suggested that the 6-hour rule between injury and surgical intervention needs to be re-evaluated^16^.

Pin site infection usually occurs in areas where there is a greater range of motion and high stress. A recent study by Ceroni *et al* suggested that excessive movement at the fixator pin-bone interface leads to pin site irritation and infection^17^. In our study, pin site infection occurred in nine patients and was managed by changing the dressing regularly. Such daily pin site care plays an important role in the treatment of pin site infections^18^.

For Ilizarov external fixation, previous recommendations included use of wires with diameter of 2mm and tension between 1,000-2,000 N. It has been shown that rigid fixation can be attained with the use of four such wires each in the proximal and distal ends of fracture. This can in turn create a stable biomechanical environment for bone formation and therefore bone union. However, due to excessive fatigue, the steel wires can break during the middle or later stages of bone transport and mineralisation^14^. Two such cases of wire breakage were also observed in this study, but, as the callus in both cases had almost reached the late mineralisation phase, there was no need to change the wire in both cases.

Non-union occurred in two of our patients with one of them undergoing amputation even after multiple procedures. Thus, Ilizarov failure was seen in two cases in our group. This failure rate was similar to that of Yin *et al* with malunion being seen in 7% and limb amputation in 4% of the patients^10^. One patient had reinfection which was treated with radical debridement and antibiotics while one patient developed septic arthritis of the knee and underwent arthrotomy, improving after the intervention. A randomised clinical trial conducted by Peres *et al* also showed arthrotomy to be an effective method for dealing with septic arthritis^19^.

All in all, in infected non-union of the tibia, union can be achieved only after control of local infection, removal of necrotic tissue from the nidus and a creation of a stable biological and biomechanical environment. The current study proves the effectiveness of Ilizarov technique for treatment of infected tibial non-union even in developing countries like Pakistan where there is considerable lack of resources and expertise. Despite the shortcomings of a resource constrained setting, success rate and complications are comparable to international literature reports.

However, the absence of any control group is a limitation of this study. In addition, outcome of this retrospective study might be underestimated due to the information bias, such as the incidence of pin tract infection (some were only grade 1-2 and were not recorded). Hence, there is still need for large scale prospective and multi-center studies, specially from developing countries to substantiate the results of the current study.

## Conclusion

Ilizarov external fixator is better suited for infected non-union of tibia because it can provide a stable mechanical environment, transport bone, correct deformities, and enable weight bearing during the course of treatment. We therefore recommend the use of Ilizarov external fixator for infected non-union of tibial fractures due to its high success rates and because it offers an opportunity to salvage the limb without eventually going for amputation. However, patient discomfort due to long-lasting treatment duration, is one of the key disadvantages of this treatment modality.
